# Risk factors associated with recurrence of Henoch–Schonlein purpura: a retrospective study

**DOI:** 10.3389/fped.2023.1164099

**Published:** 2023-06-12

**Authors:** Tongtong Cao, Hui-min Yang, Jing Huang, Yan Hu

**Affiliations:** Department of Traditional Chinese Medicine, Beijing Children's Hospital, Capital Medical University, National Center for Children's Health, Beijing, China

**Keywords:** pediatric HSP, recurrence, risk factor, renal involvement, retrospective study

## Abstract

**Background:**

Recurrence is considered a vital problem for assessing the prognosis of Henoch–Schonlein purpura (HSP). The objective of this study was to evaluate factors affecting the recurrence in children with HSP.

**Methods:**

We retrospectively reviewed records of 368 patients under the age of 16 years diagnosed with HSP from October 2019 to December 2020 in Beijing Children's Hospital. Patients were divided into a non-recurrence group and a recurrence group according to whether there was a recurrence. Incidence of manifestation, possible cause, age, and treatment were retrospectively analyzed. Univariate and multivariate logistic regression analyses were used to determine the risk factors of recurrence in HSP.

**Results:**

Percentages of patients were 65.2% for the non-recurrence group and 34.8% for the recurrence group. The percentage of patients with renal involvement was significantly higher in the recurrence group (40.6%) than in the non-recurrence group (26.3%). Respiratory tract infection was the most frequent trigger: 67.5% in the non-recurrence group and 66.4% in the recurrence group. Recurrence was more likely to occur in patients aged >6 years (53.3% *vs*. 71.9%). Logistic regression analysis revealed that hematuria plus proteinuria was an independent risk factor for the recurrence of HSP. Conversely, animal protein, exercise restriction, and age ≤6 years were independent favorable factors for the non-recurrence of HSP.

**Conclusion:**

These results suggest that organ involvement, exercise, and diet management during the initial episode of HSP should be strictly monitored for children with HSP. Adequate clinical intervention for these risk factors may limit or prevent HSP recurrence. Moreover, renal involvement is associated with the long-term prognosis of HSP.

## Background

Henoch–Schönlein purpura (HSP) is the most common vasculitis in children, with an incidence of 20/100,000 children annually (1), and is diagnosed when palpable purpura is present plus one of the following: biopsy showing predominant immunoglobulin A (IgA) deposition, arthralgia/ arthritis, abdominal cramping pain, and hematuria/proteinuria (2). HSP is considered a self-limiting disease. However, it is exceedingly subject to relapse and its recurrence is difficult to prevent. Approximately 40%–50% of patients present renal involvement and aggravate refractory HSP cases (3).

Though the exact pathogenic mechanisms of HSP are still obscure, previous studies focus on the role of several factors in predicting the recurrence of HSP. According to the statistics, approximately 30%–40% of patients with HSP have at least one recurrence during a 2-year period after the first outbreak (4). Numerous studies suggested that HSP might be associated with infection, whereas it has been stated that other factors, such as persistent rash, abdominal pain, proteinuria, hematuria, and some pathogenic microorganism, are significantly related to the recurrence of HSP (5). However, the prediction of recurrence in children has not been clearly defined yet.

## Methods

### Patients

We retrospectively reviewed the records of 368 children with HSP who were regularly consulted at Beijing Children's Hospital from October 2019 to December 2020. The patients had been diagnosed with HSP according to the 2010 European League Against Rheumatism/Pediatric Rheumatology International Trials Organization/Pediatric Rheumatology European Society (EULAR/PRINTO/PRES) criteria, were aged from 3 to 16 years and had medical history >1 year included. Most cases were diagnosed with typical clinical manifestation based on the classical diagnosis criteria, whereas some cases with HSP were confirmed by skin biopsy. Patients with skin rashes caused by other diseases combined with severe organ damage were excluded. Cases with missing key data were excluded. We conducted follow-up visits through outpatient visits and telephone contact. The follow-up period was 1 year. Patients with renal involvement and urine protein of more than 20 mg/kg/24 h and/or 20 red blood cells per high power field in the first onset were also excluded. Recurrence was defined as the presence of a fresh episode after at least 3 months without signs or symptoms (6). No recurrence was defined as a patient previously diagnosed with HSP who was in complete remission, including without rash, abdominal pain, or renal involvement for six months from the previous episode. The patients with severe gastrointestinal involvement were given low-dose steroid treatment, and those with renal involvement were administered corresponding treatment. The patients were provided with symptomatic and history support.

The epidemiological characteristics and demographic data, including age, sex, onset seasons, family history, diet history, infection, exercise, and organ involvement, were retrieved. Prescribed medications, including corticosteroids and immunosuppressors, were also recorded. Joint involvement was described as joint pain. Gastrointestinal involvement was defined as the presence of small bowel angina and postprandial abdominal pain in the context of the clinical duration of vasculitis. Edema and sub-mucosal and intramural hemorrhage under B-model ultrasonic were also defined as gastrointestinal involvement. Renal involvement was defined as hematuria and/or proteinuria. Hematuria was defined by the presence of at least five red blood cells per high-power field. Proteinuria was defined as protein loss of more than 0.3 g per 24 h. Diet restriction was prescribed; patients were recommended an animal protein-free diet before the rash completely disappeared. Exercise restriction was also prescribed; patients were recommended to avoid strenuous exercise until complete remission, including without rash, abdominal pain, or renal involvement.

### Data collection and statistical analysis

We retrieved the data, including age, sex, onset seasons, family history, diet history, infection, exercise, and organ involvement. These data were obtained from the outpatient and inpatient department. Statistical analysis was performed with the Prism software for personal computers. All continuous variables were tested for normality. Results were expressed as mean ± standard deviation or as median and range or interquartile range (IQR) as appropriate. Student’s *t*-test or Mann–Whitney *U*-test was used to compare continuous variables and the *χ*^2^ test for categorical variables. Multiple logistic regression analyses were conducted to identify the independent predictors of recurrent HSP. A *p*-value < 0.05 was considered statistically significant in all the calculations.

## Results

### Recurrent rate and clinical manifestations

A total of 368 children aged <16 years with a diagnosis of HSP were enrolled in this retrospective study between October 2019 and December 2020. The clinical characteristics of all patients are summarized in Table 1. There were 171 girls and 197 boys. The female-to-male ratio was 0.86 (46.5% female subjects and 53.5% male subjects). All patients were divided into non-recurrence and recurrence groups based on relapse/recurrence. The percentage of male/female patients showed no significant difference between the two groups (Table 1).

**Table 1:** Recurrence rate and clinical manifestations.

	Total (*n* = 368)		*χ* ^2^	*p-*value
	NR (*n* = 240)	R (*n* = 128)		
Male sex	130	67	0.112	0.738
Skin purpura only	56	21	2.421	0.120
Arthralgia/arthritis	94	32	7.855	0.005
GI involvement	110	39	8.179	0.004
Small bowel angina	103	35	8.638	0.003
Postprandial abdominal pain	34	11	2.416	0.120
Edema and sub-mucosal and intramural hemorrhage	28	12	0.453	0.501
Renal involvement	63	52	8.542	0.003
Proteinuria	18	13	0.764	0.382
Hematuria	8	6	0.418	0.518
Hematuria plus proteinuria	37	33	6.369	0.012
Renal plus GI	58	38	1.320	0.251

GI, gastrointestinal; NR, non-recurrence group; R, recurrent group.

The percentage of patients with non-recurrence was 65.2% (240/368) for the non-recurrence group and 34.8% (128/368) for the recurrence group. The percentage of patients with only skin lesions was 23.3% (56/240) for the non-recurrence group and 16.4% (21/128) for the recurrence group. The percentage of patients with arthralgia or arthritis was 39.2% (94/240) for the non-recurrence group, but it decreased in the recurrence group (25%; 32/128). Gastrointestinal involvement was observed in 110 (110/240; 45.8%) patients in the non-recurrence group, and it significantly decreased in the recurrence group (39/128; 30.5%). The percentage of patients with renal involvement was 26.3% (63/240) for the non-recurrence group and 40.6% (52/128) for the recurrence group. More specifically, the percentage of patients with renal and gastrointestinal involvement was 24.2% (58/240) for the non-recurrence group and 29.6% (38/128) for the recurrence group; there was no significant difference between the two groups (Table 1).

### Possible cause

In the non-recurrence group, 144 (60%) patients occurred with HSP in autumn and winter, and it was also high season for the recurred patients (73/128; 57%) in the recurrence group). The upper respiratory tract infection (such as pharyngalgia, fever, and cough) rate showed a similar pattern: 162/240; 67.5% in the non-recurrence group and 85/128 66.4% in the recurrence group. Moreover, it showed that pharyngalgia was the most frequent symptom of the upper respiratory tract among the two groups: 145/240; 60.4% in the non-recurrence group and 73/128; 57% in the recurrence group. Besides respiratory tract infection,17 patients (17/240; 7%) had an allergy, 14 patients (14/240; 5.8%) had taken a vaccine, and 2 patients (2/240; 0.8%) had an injury in the non-recurrence group. In the recurrence group, 12 patients (12/128; 9.4%) had an allergy, 8 patients (8/128; 6.3%) received vaccination, and no patients had an injury. It was suggested to all patients that they avoid vaccination for at least 1 year after fully recovering. However, unknown causes were found in 45 patients (45/240; 18.7%) in the non-recurrence group and in 23 patients (23/128;17.9%) in the recurrence group. There were no significant differences between the two groups for seasons and possible causes (Table 2).

**Table 2:** Seasons and possible causes for HSP.

	NR (*n* = 240)	R (*n* = 128)	*χ* ^2^	*p-*value
**Onset seasons**				
Autumn and winter	144	73	0.304	0.581
RI	162	85	0.045	0.832
Fever	14	8	0.026	0.872
Cough	43	23	0.000	0.990
Pharyngalgia	144	73	0.304	0.581
Allergy	17	12	0.604	0.437
Vaccination	14	8	0.026	0.872
Injury	2	0	0.000	1.000
Unknown cause	45	23	0.034	0.854

GI, gastrointestinal; NR, non-recurrence group; R, recurrent group; RI, respiratory tract infection.

### Renal involvement in different age groups

Table 3 shows similar proportions for age groups among 240 patients without renal involvement in the non-recurrence group (≤6 years old, 112/240, 46.6%; >6 years old, 128/240, 53.3%). However, it showed recurrence is more frequent in older patients (≤6 years old, 36/128, 28.1%; >6 years old, 92/128, 71.9%). Furthermore, among 63 patients with renal involvement in the non-recurrence group, older patients were more likely to have renal involvement: ≤6 years old, 12/63, 19% and >6 years old, 51/63, 80.9%. There was a similar trend among 52 patients with renal involvement in the recurrence group: ≤6 years old, 16/52, 30.8% and >6 years old, 36/52, 69.2% (Table 3).

**Table 3:** Renal involvement in different age groups for HSP.

	Without RI		RI	
	NR	R	NR	R
Age				
≤6years	112	36	12	16
>6years	128	92	51	36
*χ* ^2^	11.937		2.215	
*p*-value	0.001		0.145	

NR, non-recurrence group; R, recurrent group; RI, renal involvement.

### Treatment

The treatment of HSP is shown in Table 4. Patients without organ involvement were given symptomatic relief. The patients with severe gastrointestinal involvement were administered low-dose glucocorticoid treatment (1–2 mg/kg·day). Glucocorticoid was administered to 99 patients (99/240; 41.2%) in the non-recurrence group, and it was much less in the recurrence group (45/128; 35.2%), but there was no statistical difference. In the non-recurrence group, 10 patients (10/240; 4.2%) received immunoglobulin, but only 4 patients (4/128; 3.1%) with recurrence were given this treatment.

**Table 4:** Treatment for HSP.

	NR (*n* = 240)	R (*n* = 128)	*χ* ^2^	*p-*value
APR	186	53	47.769	<0.001
ER	197	42	89.015	<0.001
GC	99	45	1.301	0.254
IP	10	4	0.045	0.833

NR, non-recurrence group; R, recurrent group; APR, animal protein restriction; ER, exercise restriction; GC, glucocorticoid; IP, immunoglobulin and plasma.

All patients were given a prescription for an animal protein-restrictive diet and exercise restriction until the rash completely disappeared. There was better compliance with the animal protein restriction diet in the non-recurrence group (186/240; 77.5%) than in the recurrence group (53/128; 41.4%). The non-recurrence group also complied better with the exercise restrictions (197/240; 82.1%) than the recurrence group (42/128; 32.8%).

### Possible risk factors for recurrence

After multivariate logistic regression analysis, animal protein-restrictive diet (OR = −0.439; 95% CI: 0.241–0.798; *p* = 0.007), exercise restriction (OR = 0.155; 95% CI: 0.086–0.280; *p* < 0.001), age ≤ 6 years (OR = 0.483; 95% CI: 0.272–0.856; *p* = 0.013) were the protective factors for the recurrence of HSP. Conversely, the initial onset with hematuria plus proteinuria was found to be an independent risk factor for the recurrence of HSP (OR = 10.342; 95% CI: 3.772–28.354; *p* < 0.001) (Table 5).

**Table 5:** Possible risk factors for recurrence.

Risk factor	*β* value	SE value	Wald value	*p*-value	OR value	95% CI
HPP	2.336	0.515	20.613	<0.001	10.342	3.772–28.354
Age ≤6	−0.729	0.293	6.196	0.013	0.483	0.272–0.856
APR	−0.824	0.305	7.286	0.007	0.439	0.241–0.798
ER	−1.865	0.302	38.205	<0.001	0.155	0.086–0.280

HPP, hematuria plus proteinuria; APR, animal protein restriction; ER, exercise restriction.

## Discussion

HSP is an immune complex-mediated and self-limiting vasculitis in childhood, which affects small vessels of the skin, joints, gastrointestinal system, and kidneys. Patients with HSP may exhibit different manifestations and organ involvement (7). Studies have focused on the possible risk factors in patients with HSP and its recurrence. However, the exact risk factors in patients with different organ involvement and the recurrence of HSP have not been illustrated yet (8). Therefore, this study aimed to evaluate the characteristic type of HSP and the possible risk factor in the recurrence of HSP. Moreover, it also assessed the relationship between clinical features and the occurrence of HSP.

Our recent study analyzed the recurrence risk factor by following up with 368 patients with HSP who were regularly consulted from October 2019 to December 2020. Results showed that the infections were subjected to an obvious increase in patients with HSP compared with other causes. Renal involvement has also been shown to be involved in the recurrence of HSP. In accordance with other studies, the present study showed similar clinical characteristics of HSP with skin lesions.

Our study showed that, in the non-recurrence group, presentation with skin lesions only was 23.3%, skin plus joint was 39.2%, skin plus gastrointestinal involvement was 45.8%, and renal involvement was 26.3%. The frequency of HSP patients with gastrointestinal and renal involvement was much higher in our study. It is probable that patients with HSP who presented skin lesions only usually received treatment in local hospitals, but patients with gastrointestinal or renal injuries attended our hospital for treatment. Thus, this might explain the percentage of different types of HSP in our center. In this study, the recurrence rate of HSP in children was 34.7% (128/368), which was close to that reported in recent studies (33% recurrence rate), suggesting that the recurrence rate of HSP in children is high (9). Therefore, it is beneficial to guide the clinical treatment of children, reduce the recurrence rate, and improve the prognosis.

The manifestation of recurrent HSP is quite variable in childhood (10) and the exact incidence of the variable types in children with HSP is rarely reported. Some studies reported the percentage of recurrent HSP but rarely reported the exact types (11). Significant differences probably resulted from the recruited patients in different studies. We reviewed the patients with various manifestations, including joint, gastrointestinal, and renal involvement. Renal involvement has been reported in 20%–50% of children with HSP. Our study suggested renal damage had a higher recurrence rate than other manifestations, such as rash, arthralgia or arthritis, and gastrointestinal involvement. In the present study, the frequency of HSP was concluded to be 40.6% in the recurrence group and 26.3% in the non-recurrence group. Moreover, our results suggested that renal involvement recurred more frequently relative to joint and gastrointestinal involvement. In addition, our study indicated that renal involvement should be considered a crucial monitoring index in patients with HSP.

At the time of the diagnosis of HSP, most patients presented with skin lesions and additional symptoms, including joint, gastrointestinal, and renal involvement. As opposed to the predictor factors found, Prais D (12) reported that no clinical or laboratory characteristics were discovered to be a risk factor for predicting recurrence. This finding probably resulted from different definitions of recurrence and recruited patient selection. In the present study, initial onset with renal damage was an independent risk factor for the recurrence of HSP. It has also been reported that urine protein positivity is a risk factor for HSP recurrence (13). Therefore, there is a need for continuous monitoring and high attention in the case of kidney damage occurrence. Consistent with previous data (14, 15), the history of previous infections in our study was found to be related to the occurrence of HSP. We speculate that the reason may be that children with upper respiratory tract infections produce a variety of inflammatory mediators that change the immune balance of the body, easily leading to the occurrence of HSP.

Moreover, exercise restriction was found to be a protective factor for recurrent HSP. Histopathological features of HSP are leukocytic vasculitis of arterioles (16). Although few studies have focused on the relationship between exercise and HSP recurrence, it is not hard to speculate frequent and intense exercise accelerates the relaxation and contraction of small blood vessels between muscle tissues, increases the risk of capillary damage, and is prone to recurrence of HSP. Therefore, in the clinical treatment of HSP, children should also pay attention to controlling their exercise intensity. However, excessive restriction of physical activity will result in a decline in the immunity of children and increase the susceptibility of the body. Therefore, further research is needed to achieve a balance between exercise and immunity.

In our experience of clinical treatment of purpura, an animal protein restrictive diet is warranted, especially if a rash is present. The cutoff point for animal protein restriction is usually suggested until the rash completely disappears. Our study discovered better compliance with the animal protein-restrictive diet in the non-recurrence group (186/240; 77.5%) than in the recurrence group (53/128; 41.4%). Our results suggested that an animal protein-restrictive diet was an independent protective factor for recurrent HSP. However, most of the recurrence patients did not follow the doctor's instructions. In addition, many parents chose to maintain the animal protein restrictive diet for a long time even after rashes had disappeared, resulting in malnutrition. Moreover, there are few studies on the relationship between diet and HSP recurrence. We hope that further perspective multi-center studies will consider the role of diet management for patients with HSP.

In this study, the onset age was significantly higher in patients aged over 6 years in the recurrence group. We discovered a small proportion of HSP with renal involvement in patients aged <6 years. Furthermore, age ≤6 years was also an independent protective factor for recurrent HSP. Reasons for discrepancies in patients with different age groups could be partially associated with special immune states in different age groups. Therefore, it is significant to research immunological mechanisms in different age groups in patients with HSP.

We included patients with various manifestations, including rash, joint pain, gastrointestinal involvement, and renal injury, in the present study. However, several limitations might be seen in the present study. First, all cases came only from one center, so the study population was a small sample size, and our center received mostly patients with severe manifestations, hence resulting in uneven case distribution. In addition, we suggested that appropriate exercise and diet restrictions for patients with HSP could decrease the recurrence of HSP. However, we did not determine how long the restriction should last; there is a need for further prospective studies to define a specific point in time. We suggested renal involvement could be an independent risk factor for the recurrence of HSP, and renal involvement recurred more frequently than other types of HSP. This finding confirms that patients with HSP need preventative treatment for renal involvement. Further controlled clinical studies in a large population and multi-center follow-up should be considered.

## Data Availability

The original contributions presented in the study are included in the article, further inquiries can be directed to the corresponding author.

## References

[1] Torrelo A Noguera L. Henoch–Schönlein purpura. New York: Springer (2015).

[2] Jaszczura M Dyga K Bryłka A Góra A Machura E. Iga vasculitis, formerly known as Henoch–Schönlein purpura—the most common vasculitis in children. Pediatr Pol. (2018) 93(4):336–42. 10.5114/polp.2018.78000

[3] Barut K Sahin S Kasapcopur O. Pediatric vasculitis. Curr Opin Rheumatol. (2016) 28(1):29–38. 10.1097/BOR.0000000000000236 26555448

[4] Saulsbury FT. Henoch–Schönlein purpura. Curr Opin Rheumatol. (2001) 13:35–40. 10.1097/00002281-200101000-00006 11148713

[5] Fan GZ Li RX Jiang Q Niu MM Qiu Z Chen WX Streptococcal infection in childhood Henoch–Schönlein purpura: a 5-year retrospective study from a single tertiary medical center in China, 2015–2019. Pediatr Rheumatol. (2021) 19:79. 10.1186/s12969-021-00569-3 PMC8173722 34078391

[6] Alfredo CS Nunes NA Len CA Barbosa CMP Terreri MTRA Hilário MOE. Henoch–Schönlein purpura: recurrence and chronicity. J Pediatr (Rio J). (2007) 83(2):177–80. 10.1590/S0021-75572007000200013 17327931

[7] Brown Patrick J Haught Justin M English Joseph C. Periumbilical purpura prior to gastrointestinal involvement in Henoch–Schönlein purpura. Am J Clin Dermatol. (2009) 10:127–30. 10.2165/00128071-200910020-00006 19222253

[8] García-Porrúa C González-Louzao C Llorca J González-Gay MA. Predictive factors for renal sequelae in adults with Henoch–Schönlein purpura. J Rheumatol. (2001) 28(5):1019–24. 11361182

[9] Mahmood L Zulfiqar F Khalid S Dillon M. P200 management of Henoch–Schönlein purpura (HSP). Arch Dis Child. (2019) 104(Suppl 3):A238. 10.1136/ARCHDISCHILD-2019-EPA.555

[10] Pan YX Ye Q Shao WX Shang SQ Mao JH Zhang T Relationship between immune parameters and organ involvement in children with Henoch–Schonlein purpura. PLoS One. (2014) 9(12):e115261. 10.1371/journal.pone.0115261 25514176 PMC4267823

[11] Trapani S Micheli A Grisolia F Resti M Chiappini E Falcini F Henoch–Schönlein purpura in childhood: epidemiological and clinical analysis of 150 cases over a 5-year period and review of literature. Semin Arthritis Rheum. (2005) 35(3):143–53. 10.1016/j.semarthrit.2005.08.007 16325655

[12] Dario P Jacob A Moshe N. Recurrent Henoch–Schönlein purpura in children. J Clin Rheumatol. (2007) 13:25–8. 10.1097/01.rhu.0000255692.46165.19 17278945

[13] Lei WT Tsai PL Chu SH Kao YH Lin CY Fang LC Incidence and risk factors for recurrent Henoch–Schönlein purpura in children from a 16-year nationwide database. Pediatr Rheumatol. (2018) 16(1):25. 10.1186/s12969-018-0247-8 PMC5902957 29661187

[14] Jauhola O Ronkainen J Koskimies O Ala-Houhala M Arikoski P Hölttä T Clinical course of extrarenal symptoms in Henoch–Schonlein purpura: a 6-month prospective study. Arch Dis Child. (2010) 95(1):871–6. 10.1136/adc.2009.167874 20371584

[15] Terraneo L Lava SA Camozzi P Zgraggen L Simonetti GD Bianchetti MG Unusual eruptions associated with mycoplasma pneumoniae respiratory infections: review of the literature. Dermatology. (2015) 231(2):152–7. 10.1159/000430809 26067570

[16] Yiannias JA el-Azhary RA Gibson LE. Erythema elevatum diutinum: a clinical and histopathologic study of 13 patients. J Am Acad Dermatol. (1992) 26:38–44. 10.1016/0190-9622(92)70003-X 1732334

